# Perivascular epitheloid cell tumour (PEComa) of the retroperitoneum – a rare tumor with uncertain malignant behaviour: a case report

**DOI:** 10.1186/1752-1947-3-62

**Published:** 2009-02-16

**Authors:** Alexandra M Koenig, Alexander Quaas, Thorsten Ries, Emre F Yekebas, Karim A Gawad, Yogesh K Vashist, Christoph Burdelski, Oliver Mann, Jakob R Izbicki, Andreas Erbersdobler

**Affiliations:** 1 Department of General, Visceral and Thoracic Surgery, University Medical Centre of Hamburg-Eppendorf, Martinistraße 52, Hamburg, Germany; 2 Institute of Pathology, University Medical Centre of Hamburg-Eppendorf, Martinistraße 52, Hamburg, Germany; 3 Department of Diagnostic and Interventional Radiology, University Medical Centre of Hamburg-Eppendorf, Martinistraße 52, Hamburg, Germany

## Abstract

**Introduction:**

Perivascular epitheloid cell tumours are rare mesenchymal neoplasms characterized by a proliferation of perivascular cells with an epitheloid phenotype and expression of myomelanocytic markers.

**Case presentation:**

Here we present the case of a cystic perivascular epitheloid cell tumour of the retroperitoneum associated with multifocal lung lesions. A 27-year-old woman underwent laparotomy to remove a 10 × 6 × 4 cm sized retroperitoneal mass. The resected specimen was subjected to frozen and permanent histological sections with conventional and immunohistochemical stains, including antibodies against HMB45. The tumour displayed the typical morphological and immunohistochemical features of a perivascular epitheloid cell tumour. Focal necrosis and a proliferative index of 10% suggested a malignant potential. Moreover, postoperative computed tomography scans demonstrated multiple lung lesions, which were radiologically interpreted as being most likely compatible with lymphangioleiomyomatosis.

**Conclusion:**

Since lymphangioleiomyomatosis, an otherwise benign condition, belongs to the family of perivascular epitheloid cell tumours, it cannot be excluded that the lung lesions in this case in fact represent metastases from the retroperitoneal perivascular epitheloid cell tumour rather than independent neoplasms. More experience with this new and unusual tumour entity is clearly needed in order to define reliable criteria for benign or malignant behaviour.

## Introduction

Perivascular epitheloid cell tumours (PEComas) are mesenchymal tumours composed of distinctive, so-called perivascular epitheloid cells, which were first described by Bonetti in 1992 and were observed in "sugar tumours" of the lung as well as in angiomyolipomas of the kidney [1]. These cells are characterized by an epitheloid shape, eosinophilic cytoplasm, perivascular location and a coexpression of immunohistochemical markers indicating both smooth muscle and melanocytic differentiation. PEComas are related to the tuberous sclerosis complex (TSC), characterized by mental retardation, seizures and cellular proliferations. The PEComa family includes angiomyolipomas, clear cell "sugar" tumours of the lung, pancreas and uterus and lymphangioleiomyomatosis (LAM) [2,3]. The latter is a rare disease, which typically manifests as multiple lung lesions in young women consisting of tumour-like proliferations of lymphatic channels and smooth muscle cells. Although considered a benign tumour-like lesion, LAM may lead to a rapid deterioration of lung function and the need for lung transplantation.

There are some important open questions about PEComas: the histogenesis, the normal counterpart of PEC and the identification of the histological criteria of malignancy.

We report the unusual case of a patient with a malignant retroperitoneal PEComa and subsequent detection of multiple lung lesions compatible with LAM.

## Case presentation

**Figure 1:**
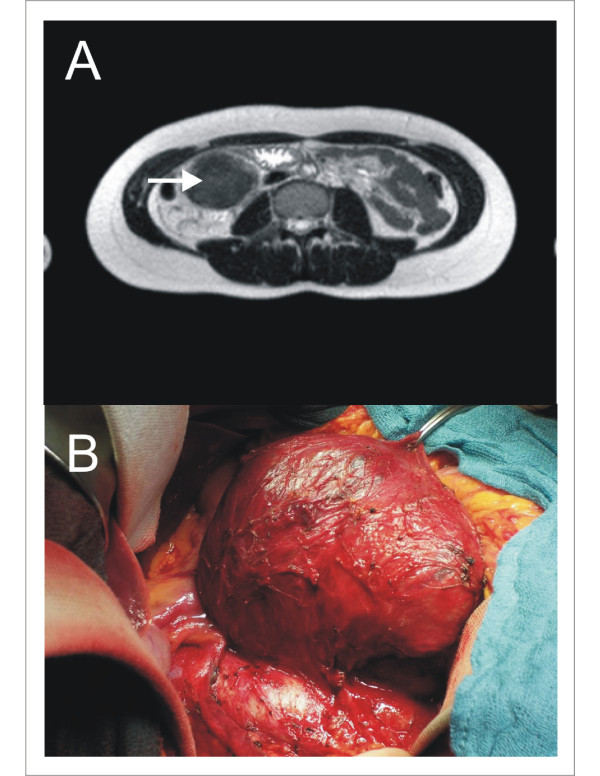
**Macroscopic Tumour**. **A**: MRT revealed the presence of a large, well circumscribed right sided retroperitoneal mass measuring 10 × 8 cm in size. **B**: Macroscopically the 10 × 6 × 4 cm sized tumour was soft with focal areas of haemorrhage circumscribed by a 2,5 × 1,5 × 0,3 cm sized capsule and with central necrosis.

**Figure 2:**
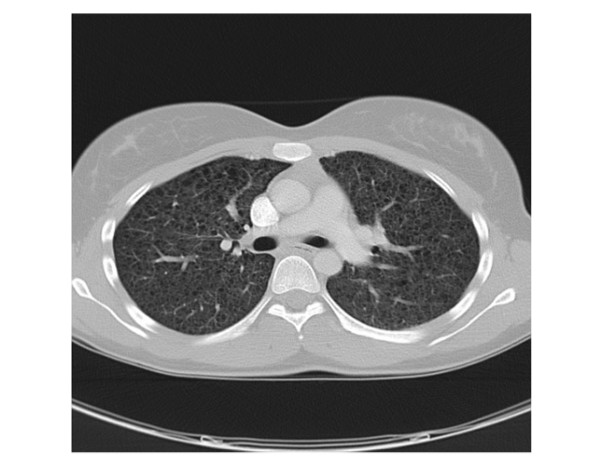
**Postoperative Chest CT Scan**. A chest CT scan showing diffuse small thin walled cystic lesions in the parenchyma of both lungs.

A 27-year-old woman, who first complained of upper abdominal pain, was referred from a local clinic with the impression of a retroperitoneal haematoma after blunt abdominal trauma 4 months ago. Magnetic resonance tomography (MRT) of the abdomen revealed the presence of a large, well-circumscribed, right-sided retroperitoneal mass measuring 10 × 8 cm in size with an irregular echogenicity (Figure 1A). The mass compressed the right kidney and the caval vein without renal involvement. No chylous ascites was present. No clinical evidence of tuberous sclerosis was present and there was no family history of cancer or known genetic disorders. Based on the MRT, the retroperitoneal mass was removed completely by laparotomy (Figure 1B). During the postoperative course the patient complained of exertional dyspnoea. The subsequently performed computed tomography (CT) scan of the lung showed the typical image of LAM with numerous thin-walled cysts throughout both lungs, but without spontaneous pneumothorax or chylous pleural effusions (Figure 2).

**Figure 3:**
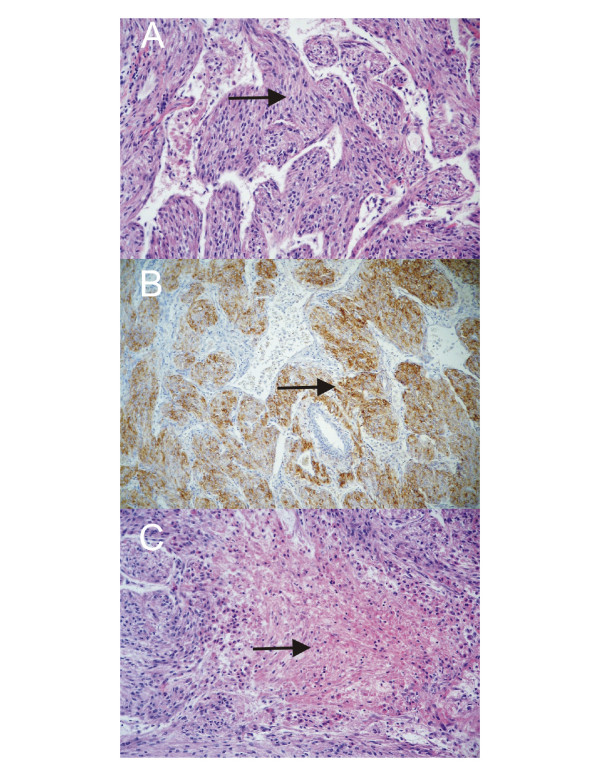
**Histological Findings**. **A**: The perivascular epitheloid cells proliferate haphazardly around slit-like vascular channels, with aggregation of lymphoid cells. **B**: Tumour cells with expression of the HMB45-antigen. (immunohistochemistry with the avidin-biotin-peroxidase-complex method; counterstain haematoxylin; original magnification × 100). **C**: Focal coagulative necrosis of tumour cells (H&E; original magnification × 200).

Macroscopically, the 10 × 6 × 4 cm sized mass had a soft consistency and was circumscribed, but not truly encapsulated. On cut sections, large, central areas of haemorrhage could be observed, giving it an impression of an old haematoma. On frozen sections, it became obvious that the wall of the cystic mass consisted of nests of tumour cells with a uniform, spindle shape. There were no overt signs of malignancy. On permanent sections, the tumour displayed fascicles and nests of elongated epitheloid tumour cells with a clear to pale eosinophilic cytoplasm, arranged around numerous ectatic blood vessels (Figure 3A). Sometimes, the tumour cell proliferations seemed to evolve directly from the walls of medium-sized blood vessels. Occasional mitoses and foci of haemorrhage and necrosis were present (Figure 3C). Immunohistochemically, most of the tumour cells showed a positive reaction for alpha-smooth muscle actin (SMA), Desmin and HMB45 (Figure 3B). About 50% of tumour cells showed a weak positivity for the oestrogen receptor. Proliferative activity, as measured by an antibody against the Ki-67 antigen, was 10% of tumour cells. Cytokeratins, epithelial membrane antigen (EMA), synaptophysin, S100, and CD117 (c-kit) were negative. CD31, CD34 and D2-40 decorated the endothelial linings of the numerous vessels. The diagnosis of a tumour with perivascular epitheloid cell differentiation, a so-called PEComa, was made. Based on the histological findings on permanent sections, a malignant potential was suggested. The margins of resection were free of tumour cells.

## Discussion

Neoplasms with perivascular epitheloid cell differentiation are a group of ubiquitous mesenchymal tumours sharing morphological, immunohistochemical, ultrastructural and genetically distinctive features [4]. These PEComas are characterized by cells with an epitheloid appearance, a clear to eosinophil cytoplasm and an intimate relationship to blood vessels [5]. The cells are consistently immunoreactive to the melanocytic marker HMB45, variably immunoreactive to smooth muscle actin and negative for epithelial markers. The histogenesis and physiological counterparts of PEC are unknown. The PEComa family comprises angiomyolipomas (AML), clear cell "sugar" tumour of the lung (CCST), lymphangioleiomyomatosis (LAM), clear cell myomelanocytic tumour of the falciform ligament/ligamentum teres (CCMMT) and unusual clear cell tumours of the pancreas, rectum, abdominal serosa, uterus, vulva, thigh and heart. The uterus is one of the most prevalent sites of involvement [6].

Clinically, a subset of PEComas behaves in a malignant fashion. Clear criteria for malignancy have not been elaborated in this very rare tumour entity until now, owing to their rarity. Folpe et al. reported 26 cases of PEComas of soft tissue and gynaecological origin proposing criteria for the classification of these tumours as "benign", "of uncertain malignant potential" and "malignant". In our patient we observed a significant association between tumour size >5 cm, infiltrative growth pattern, high nuclear grade, necrosis and mitotic activity >1/50 HPF and subsequent aggressive clinical behaviour of PEComas [7]. Surgery seems to be the only approach for aggressive cases, as chemo- and radiotherapy have not shown significant results.

The above reported tumour is a rare case of a PEComa arising in the retroperitoneum.

Based on the occasional foci of necrosis, the infiltrative growth pattern on microscopic level, as well as the relatively high proliferative activity suggested a malignant potential in the present case. Even more unusual is the subsequent occurrence of multiple pulmonary lesions, which were radiologically described as being quite typical for lymphangioleiomyomatosis (LAM). This rare disease usually occurs in young women of childbearing age and is characterized by a distinctive proliferation of lymphatic and smooth muscle cells. The primary site of origin is the lung and occurrence is usually associated with decreased pulmonary function and chylous effusions [8]. The speculation that LAM is a female sex hormone dependent tumour is supported by the high prevalence rate in women of reproductive age and exacerbation of the disease in pregnancy. Several studies regarding clinical trials of hormonal therapy have been reported [9,10]. Surgical intervention is necessary in complications (thoracic drainage, pleurectomy for recurrent pneumothorax). If hormonal therapy is not successful, a combined heart and lung transplantation should be attempted as ultima ratio [11]. LAM can occur without evidence of other disease (sporadic LAM) or in conjunction with tuberous sclerosis complex, an autosomal dominant tumour suppressor gene syndrome characterized by seizures, mental retardation, and tumours in the brain, heart, skin and kidney.

Therefore, a full work up for tuberous sclerosis is necessary in these patients.

The association between LAM and PEComas as a family and the co-occurrence in an individual patient is well known. It could be speculated that these patients may have a special predisposition to develop such tumours. On the other hand, it cannot be excluded that the pulmonary lesions actually represent metastases from the retroperitoneal PEComa. However the possibility that the lung lesions represent metastases is doubtful. It is most likely that they represent separate lesions as true LAM.

Since the pulmonary lesions were not biopsied, a histological comparison to the retroperitoneal tumour was not possible. However, it is likely that even histological examination of the pulmonary tumours would not have been able to solve the question of metastatic versus independent origin, since both metastasis of a PEComa and primary LAM could have the same histological appearance. Clinical follow-up must show if the pulmonary lesions will behave in the typical fashion of LAM.

## Conclusion

This case report demonstrates the diagnostic, prognostic and therapeutic dilemmas of a new and rare tumour entity. The outcome of this disease can be devastating, yet the aetiology and effective treatments are unknown. Firm criteria for malignancy and proper subclassifications of PEComas have yet to be established and should be validated by case reports and studies of clinical behaviour.

## Abbreviations

AML: angiomyolipoma; CCMMT: clear cell myomelanocytic tumour of the falciform ligament/ligamentum teres; CCST: clear cell "sugar" tumour of the lung; CT: computed tomography; EMA: epithelial membrane antigen; LAM: lymphangioleiomyomatosis; MRT: magnetic resonance tomography; PEC: perivascular epitheloid cell tumor; SMA: alpha-smooth muscle actin; TSC: tuberous sclerosis complex

## Consent

Written informed consent was obtained from the patient for publication of this case report and accompanying images. A copy of the written consent is available for review by the Editor-in-Chief of this journal.

## Competing interests

The authors declare that they have no competing interests.

## Authors' contributions

AMK initiated the concept, literature search and write up of the manuscript. AQ performed the pathological investigations and helped in the literature search. TR performed and diagnosed the CT scans. EFY helped in revision of the article. KAG contributed to the clinical management of the patient and gave approval for the final write up. YKV assisted in performing the surgery and helped in drafting the article. CB helped in the revision of the article. OM performed the surgery. JRI is the consultant surgeon responsible for the patient's care and made final corrections to the manuscript. AE diagnosed the specimens and supervised the overall structure of the article. All authors read and approved the final manuscript.
